# Pre-Implant Substrate, Peri-Implant Electrical Features, and Outcomes Across Four Echocardiographic Phenotypes After CRT-D

**DOI:** 10.3390/jcm15197443

**Published:** 2026-09-24

**Authors:** Vasileios Cheilas, Anna Kostopoulou, Giorgos Filandrianos, Emmanuel M. Kanoupakis, George Poulos, Emmanuel Koutalas, Athanasios Saplaouras, Panagiotis Mililis, Michalis Efremidis, Giorgos Tsironis, Maria Marketou, Konstantinos P. Letsas, George E. Kochiadakis

**Affiliations:** 1 School of Medicine, University of Crete, 70013 Heraklion, Greece; 2 Onassis Cardiac Surgery Center, 17674 Athens, Greece; kostopoulou@ocsc.gr (A.K.);; 3 Artificial Intelligence and Learning Systems Laboratory, National Technical University of Athens, 15780 Athens, Greece; 4 University Hospital of Heraklion, 71500 Heraklion, Greece; 5 Department of Physics, University of Crete, 70013 Heraklion, Greece

**Keywords:** CRT-D, reverse remodeling, left ventricular end-systolic volume, appropriate ICD shock, heart failure hospitalization, risk stratification

## Abstract

**Background:** Reverse remodeling after cardiac resynchronization therapy with defibrillator backup (CRT-D) is heterogeneous and may reflect both pre-implant substrate and post-implant electrical response, with implications for subsequent outcomes. **Objectives:** This study evaluated a graded echocardiographic response framework after CRT-D as an integrative phenotype linking pre-implant substrate, peri-implant electrical features, and post-landmark clinical outcomes. **Methods:** The analytic cohort included 334 CRT-D recipients with complete four-group classification based on relative left ventricular end-systolic volume reduction at the first eligible post-implant echocardiogram. Baseline correlates of the 4-class response phenotype were assessed using ordinal logistic regression, supported by permutation-importance analysis. A stacked peri-implant model added ΔQRS, RV1SI pattern on ECG, left ventricular lead position, and lead depth. Time-to-event analyses used a landmark design anchored at the first post-CRT echocardiogram. **Results:** In the pre-implantation ordinal model, female sex (*p* = 0.005), higher baseline left ventricular ejection fraction (*p* = 0.032), left bundle branch block (*p* = 0.038), and dilated cardiomyopathy (*p* = 0.048) were associated with a more favorable response class. In the stacked peri-implant model, ΔQRS was the strongest correlate of higher response class (OR: 1.41; 95% CI: 1.15–1.73; *p* = 0.0008). Mortality differed across response classes (log-rank *p* < 0.001); compared with super-responders, negative responders had higher adjusted mortality (HR: 4.41; 95% CI: 1.82–10.70). At 60 months, heart failure hospitalization ranged from 23.3% to 2.9% and first appropriate ICD shock from 29.3% to 9.0% from negative responders to super-responders. **Conclusions:** After CRT-D, reverse-remodeling phenotype identified clinically distinct post-landmark risk profiles. Less favorable remodeling was associated with worse mortality, heart failure hospitalization, and appropriate ICD shock.

## 1. Introduction

Cardiac resynchronization therapy with defibrillator backup (CRT-D) is an established treatment for selected patients with heart failure, reduced left ventricular systolic function, and electrical dyssynchrony [1,2]. In appropriately selected patients, CRT improves symptoms, promotes reverse left ventricular remodeling, and reduces heart failure hospitalization and mortality [1,2]. Yet response remains heterogeneous, with 30% to 50% of patients categorized as nonresponders [3].

CRT response has been defined inconsistently across the literature, and no single standard has been universally adopted [4]. In a widely cited analysis, Fornwalt et al. showed that 26 highly cited CRT studies used 17 different primary response criteria, with poor agreement among alternative definitions [4]. Ypenburg et al. subsequently proposed an echocardiographic classification based on the magnitude of left ventricular end-systolic volume reduction—negative responders, nonresponders, responders, and super-responders—and demonstrated that greater reverse remodeling was associated with more favorable long-term prognosis [5]. Consistent with this, the REVERSE analysis showed that patients who worsened after CRT had high mortality, whereas those who stabilized early had a substantially better prognosis than conventional nonresponder classifications would suggest [6]. Together, these observations suggest that CRT response is better viewed along a spectrum than as a binary label.

A useful response taxonomy should do more than classify responders and nonresponders; it should identify categories that reflect subsequent clinical trajectory. This is clinically important because the response achieved after CRT reflects the combined effects of baseline substrate and peri-implant factors, and is itself linked to subsequent hard outcomes, including mortality, heart failure hospitalization, and ventricular arrhythmic or appropriate ICD events [7]. Predictors of CRT response therefore span both patient substrate and the quality of delivered resynchronization. Favorable baseline correlates include female sex [8], nonischemic substrate [8], left bundle branch block morphology [8], and QRS duration >150 ms [8], whereas atrial fibrillation [9], baseline right ventricular dysfunction [10], and renal dysfunction [11] have been associated with less favorable response. Procedural and electrical markers, including acute QRS narrowing after implantation [12], an RV1SI post-implant ECG pattern [13], and optimal pacing site and AV delay [14], have also been linked to better echocardiographic and/or clinical response. Based on this background, we sought to evaluate CRT-D response using a four-class reverse-remodeling classification, to identify variables associated with higher response class, and to determine whether these response categories translate into distinct subsequent clinical outcomes.

## 2. Methods

### 2.1. Study Design and Population

We performed a retrospective, single-center cohort study at Onassis Cardiac Surgery Center including consecutive adult patients who underwent de novo cardiac resynchronization therapy with defibrillator backup (CRT-D) implantation at our institution between 2010 and 2025. Eligible patients were aged ≥18 years, had either ischemic or dilated cardiomyopathy, and had a baseline transthoracic echocardiographic study performed at our center within 1 year before implantation, as well as a follow-up echocardiographic study performed at our center within 1 year after implantation. Complete post-implant device follow-up, including interrogation data recorded at our institution, was also required. The follow-up echocardiographic assessment was performed at a mean of approximately 6 months after implantation.

Patients were excluded if they underwent CRT-D upgrade procedures rather than de novo implantation, if the device had been implanted at another institution, if they had cardiomyopathy subtypes other than ischemic or dilated cardiomyopathy (the analytic cohort therefore comprised only these two substrate categories, with dilated cardiomyopathy not further etiologically subclassified in the retrospective dataset), or if baseline or follow-up echocardiographic data were missing or inadequate for analysis. Patients with incomplete device follow-up or unavailable interrogation data at our center were also excluded. The derivation of the study population is summarized in Figure 1. Of 542 CRT-D cases screened during the study period, 208 were excluded according to the prespecified criteria, resulting in a final analytic cohort of 334 patients. The study was conducted in accordance with the Declaration of Helsinki and was approved by the Institutional Ethics Committee of Onassis Cardiac Surgery Center (approval no. 803/2024).

### 2.2. Data Collection and Follow-Up

Baseline data included demographic, clinical, electrocardiographic, echocardiographic, and treatment-related variables available in the retrospective dataset. These included heart failure substrate (ischemic or dilated cardiomyopathy), age at CRT-D implantation, sex, timing of baseline echocardiographic assessment, baseline left ventricular ejection fraction (LVEF),baseline left ventricular end-systolic volume (LVESV), history of PCI, history of CABG, hypertension, diabetes mellitus, hyperlipidemia, atrial fibrillation status/type, baseline conduction pattern, QRS duration before CRT-D implantation, and baseline NYHA functional class. Hyperlipidemia was treated as the pre-existing binary comorbidity recorded in the retrospective dataset and was not redefined using a study-specific total-cholesterol threshold.

Echocardiographic variables, including LVEF and LVESV, were retrospectively obtained from the official clinical echocardiography reports available in the institutional medical record. Echocardiographic images were not re-analyzed specifically for the purposes of the present study. The retrospective dataset did not contain sufficient examination-level information to reliably reconstruct either the method used for LV volume calculation or the echocardiographic software/platform across the entire study period. Formal study-specific image-quality scoring or adjudication criteria were not available; examinations with missing or inadequate echocardiographic data were excluded according to the prespecified eligibility criteria. No study-specific core-laboratory re-analysis or blinded image re-reading was performed. Because the analysis relied on routine clinical reports rather than a dedicated study-reading protocol, the number and identity of interpreting readers were not systematically captured, and operator-specific experience was likewise unavailable. Formal intraobserver and interobserver reproducibility data were therefore not available.

Myocardial deformation parameters, including LV global longitudinal strain (LV-GLS), were not systematically available across the study period and were therefore not included in the present analysis. Peri-implant variables recorded in the dataset included QRS duration after CRT-D implantation, RV1SI post-implant ECG pattern, LV lead position and LV lead depth. Additional electrical variable was ΔQRS, defined as pre-implant minus post-implant QRS duration.

Heart failure pharmacotherapy at discharge was recorded by drug class, including beta-blockers, ACE inhibitors, ARBs, ARNIs, mineralocorticoid receptor antagonists, SGLT2 inhibitors, and loop diuretics. Four-pillar therapy was defined descriptively as concurrent treatment with a beta-blocker, an ACE inhibitor/ARB/ARNI, a mineralocorticoid receptor antagonist, and an SGLT2 inhibitor. Because medication status was recorded at discharge after CRT-D implantation, these variables were summarized descriptively and were not entered into the prespecified predictive models, which were designed to evaluate pre-implant predictors (Model 1) and peri-implant electrical and device-related features (Model 2).

Follow-up variables were derived from the first eligible echocardiographic examination performed within 1 year after CRT-D implantation -landmark- (mean interval 6 months). Variables derived from this examination included follow-up left ventricular ejection fraction (LVEF), follow-up left ventricular end-systolic volume (LVESV), absolute change in LVEF, and relative reduction in LVESV.

Clinical follow-up variables included first appropriate ICD shock, first heart failure hospitalization after discharge, LVAD implantation, heart transplantation, all-cause death, and the corresponding time-to-event variables recorded in months. Follow-up was ascertained through systematic review of clinical records and sequential review of all available device interrogations at our center from implantation through the last available follow-up, ensuring continuous coverage of the intervals between successive interrogations without missing gaps in device follow-up.

### 2.3. Definition of Reverse-Remodeling Response Phenotype

The primary mechanistic endpoint of the study was a four-class reverse-remodeling response phenotype defined according to the relative change in left ventricular end-systolic volume (LVESV) between baseline and follow-up echocardiography. Response was classified using the first eligible follow-up echocardiogram obtained within 1 year after CRT-D implantation. Patients were categorized as negative responders if LVESV worsened or showed <0% reduction, nonresponders if LVESV reduction was 0% to 14.9%, responders if LVESV reduction was 15.0% to 29.9%, and super-responders if LVESV reduction was ≥30.0% [5]. This graded classification was selected to preserve the biological continuum of reverse remodeling and to avoid arbitrary dichotomization of treatment response.

### 2.4. Primary Study Objectives

The primary study objectives were: (1) to identify pre-implant variables associated with higher response class using a baseline model (Model 1) and (2) to assess the additional contribution of peri-implant variables beyond baseline information using a second model (Model 2) that incorporated the output of Model 1 together with prespecified peri-implant variables.

For the primary analyses, Model 1 was based on prespecified pre-implant variables, including heart failure substrate, age at implantation, sex, type of atrial fibrillation, baseline conduction pattern, QRS duration before CRT-D implantation, and baseline LVEF. Variables without a clear mechanistic rationale for CRT response were not included, in order to preserve parsimony and limit noise in the model. Model 2 evaluated the additional contribution of prespecified peri-implant/post-implant variables, including post-implant QRS-derived measures (including ΔQRS), RV1SI post-implant ECG pattern, LV lead position, and LV lead depth, in combination with the output of Model 1.

### 2.5. Secondary Prognostic Analyses

Secondary analyses assessed the prognostic significance of the 4-class response phenotype by examining the relationship between response category and subsequent adverse clinical outcomes. These outcomes included all-cause death, first heart failure hospitalization, time to first appropriate ICD shock, LVAD implantation, and heart transplantation, and each was analyzed across the 4 prespecified response categories (negative responder, nonresponder, responder, and super-responder).

### 2.6. Landmark Definition for Time-to-Event Analyses

Because response phenotype was determined using post-implant follow-up echocardiography, time-to-event analyses for secondary prognostic outcomes were performed using a landmark approach, with time zero defined as the date of the follow-up echocardiogram used for response classification. Patients were followed from this landmark date until the first occurrence of the outcome of interest or censoring at the last available device follow-up. This approach was chosen to reduce bias arising from classification of response on the basis of post-implant information.

### 2.7. Statistical Analysis

The primary echocardiographic endpoint was CRT response classified as a 4-level ordinal phenotype by relative LVESV reduction at the first post-CRT echocardiographic assessment: negative responder (<0%), nonresponder (0% to 14.9%), responder (15.0% to 29.9%), and super-responder (≥30%). To assess the sensitivity of the findings to classification near the prespecified LVESV boundaries, a sensitivity analysis was performed after excluding patients whose relative LVESV reduction was within ±1 percentage point of the 0%, 15%, or 30% thresholds. To evaluate the potential influence of variation in follow-up echocardiographic timing, a sensitivity analysis was performed after restricting the cohort to patients whose response-defining echocardiogram was obtained between 3 and 9 months after CRT-D implantation. Pre-implantation correlates were evaluated using ordinal logistic regression, supported by descriptive trend analyses. The proportional-odds assumption underlying the ordinal logistic regression was formally assessed. Because the global assessment indicated evidence of departure from proportional odds, threshold-specific cumulative logistic models were additionally examined as a sensitivity analysis. Pooled ordinal odds ratios were therefore interpreted as summary associations with progression toward a more favorable response phenotype rather than as strictly threshold-invariant effects. Potential nonlinear relationships of continuous predictors were assessed using natural cubic splines with 3 degrees of freedom, with likelihood-ratio tests comparing spline and linear specifications. Multicollinearity was assessed using variance inflation factors (VIFs), with factor-adjusted generalized VIFs used for multilevel categorical predictors where appropriate. Variables entering the final ordinal models were complete; therefore, no statistical imputation was required. In addition to overall ROC-AUC and balanced accuracy, predictive performance was evaluated using pooled out-of-fold probabilities from the 5-fold stratified cross-validation procedure. Multiclass Brier scores, class-specific one-vs-rest ROC-AUC, precision, recall, F1 score, calibration-in-the-large, calibration slopes, and confusion matrices were calculated. Continuous predictors were prescaled for interpretability: age per 10-year increase, baseline LVEF per 5-percentage-point increase, and baseline QRS duration per 10 ms increase. To complement inference with internally validated predictive assessment, 5-fold stratified cross-validated L1-regularized multiclass models were fit, with performance summarized using weighted 1-vs-rest area under the receiver operating characteristic curve and balanced accuracy; variable relevance was explored using permutation importance. A stacked peri-implantation model combined out-of-fold predicted class probabilities from the pre-implantation model with ΔQRS, post-CRT V1-positive/I-negative pattern, LV lead position, and LV lead depth. Cross-validation folds were generated using stratified 5-fold splitting with shuffling and a fixed random seed was used. Preprocessing and L1 regularization were performed within the cross-validation pipelines, while the predictor set was prespecified rather than selected through data-driven screening. For Model 2, each patient’s Model 1 probabilities were obtained out-of-fold from a model that had not been trained on that patient, and three nonredundant class probabilities were used as second-stage predictors.

ΔQRS was expressed per 10 ms increase. Time-to-event analyses used a landmark design, with time zero defined at the first post-CRT echocardiographic assessment. All-cause mortality was analyzed using Kaplan–Meier methods, log-rank testing, and Cox regression. For first heart failure hospitalization and first appropriate ICD shock, cumulative incidence functions were used because death could preclude event observation, and parsimonious cause-specific Cox models were used for adjusted analyses. LVAD implantation and heart transplantation were treated as follow-up-truncating interventions. Patients with pre-landmark events were excluded only from the corresponding endpoint-specific analysis. Primary adjusted mortality models included response group, age, sex, heart failure substrate, baseline LVEF, baseline NYHA class, atrial fibrillation type, baseline QRS duration, diabetes, and hypertension. More parsimonious models were used for heart failure hospitalization and shock because of lower event counts. All tests were 2-sided, and a *p* value <0.05 was considered statistically significant. Statistical analyses were performed using Python 3.10.12 (pandas, NumPy, statsmodels, scikit-learn, and matplotlib), with custom scripts developed for landmark time-to-event derivation and internal cross-validation.

## 3. Results

### 3.1. Study Population and Response-Class Distribution

After application of the prespecified eligibility criteria and analytic preprocessing steps, the final study cohort included 334 patients. The four-class reverse-remodeling response phenotype showed a relatively balanced distribution across categories: 63 negative responders (18.9%), 90 nonresponders (26.9%), 103 responders (30.8%), and 78 super-responders (23.4%). The median timing of the response-defining follow-up echocardiogram was 7 months (IQR 3–12) in negative responders, 5 months (IQR 3–8.75) in nonresponders, 6 months (IQR 2–10) in responders, and 6 months (IQR 2–12) in super-responders, with no statistically significant difference across response groups (Kruskal–Wallis *p* = 0.240). In a sensitivity analysis restricted to examinations performed 3–9 months after implantation (*n* = 144), the direction of the principal baseline associations remained consistent with the primary analysis. Female sex and LBBB remained statistically significant, whereas baseline LVEF and dilated cardiomyopathy showed concordant effect directions but reduced statistical precision in the smaller cohort. As shown in Table 1, the cohort had a mean age of 65.3 ± 11.1 years, and 21.6% were women. Dilated cardiomyopathy was the predominant heart failure substrate (58.4%), whereas 41.6% had ischemic cardiomyopathy. Hypertension, hyperlipidemia, and diabetes mellitus were present in 84.1%, 69.2%, and 35.3% of patients, respectively. Atrial fibrillation was absent in 47.9% of patients, while 23.7% had permanent and 28.4% had paroxysmal atrial fibrillation. Baseline LBBB was present in 79.3%, mean QRS duration before implantation was 157.6 ± 25.3 ms, and mean baseline LVEF was 28.8 ± 5.4%. Most patients were in NYHA functional class III at implantation (84.4%), consistent with an advanced heart failure population undergoing CRT-D therapy. At discharge, 324 patients (97.0%) were receiving a beta-blocker, 284 (85.0%) an ACE inhibitor/ARB/ARNI, 285 (85.3%) a mineralocorticoid receptor antagonist, 154 (46.1%) an SGLT2 inhibitor, and 272 (81.4%) a loop diuretic. Overall, 121 patients (36.2%) were receiving all four components of contemporary foundational HFrEF therapy.

For the primary pre-implant analysis, the final baseline model was constructed using seven prespecified variables: heart failure substrate, age, sex, baseline LVEF, atrial fibrillation with type, baseline conduction pattern, and QRS duration before CRT-D implantation. These variables formed the basis of Model 1, which was designed to evaluate baseline determinants of higher response class.

For the peri-implant analysis, Model 2 was built by combining the out-of-fold class probabilities generated by Model 1 with prespecified peri-implant variables, including ΔQRS, positive V1/negative I pattern after CRT, LV lead position, and LV lead depth.

### 3.2. Pre-Implant Determinants of Response Phenotype: Model 1

In the pre-implant analysis, ordinal logistic regression identified several baseline variables associated with higher response class. Female sex was associated with a higher likelihood of belonging to a more favorable response category (OR: 1.35; 95% CI: 1.09–1.67; *p* = 0.005), as was higher baseline LVEF (OR: 1.26; 95% CI: 1.02–1.56; *p* = 0.032). Baseline LBBB was also associated with higher response class (OR: 1.44; 95% CI: 1.02–2.02; *p* = 0.038), as was dilated cardiomyopathy relative to ischemic substrate (OR: 1.24; 95% CI: 1.00–1.54; *p* = 0.048). Absence of atrial fibrillation showed a borderline association with more favorable response (OR: 1.25; 95% CI: 0.97–1.63; *p* = 0.089). By contrast, age, paroxysmal atrial fibrillation, absence of bundle branch block, and baseline QRS duration were not significantly associated with response class in the final ordinal model. As detailed in Table 2, the strongest baseline correlates of a more favorable reverse-remodeling phenotype were female sex, higher baseline LVEF, baseline LBBB, and dilated cardiomyopathy substrate. These findings were also reflected in descriptive trend analyses across the four echocardiographic response phenotypes. Baseline LVEF differed across response groups, with higher median values in responders and super-responders than in negative responders and nonresponders (*p* for trend = 0.0020) (Figure 2A). Likewise, the prevalence of LBBB increased across response categories (*p* for trend = 0.0234) (Figure 2B), whereas female sex (*p* for trend = 0.0002) (Figure 2C) and dilated cardiomyopathy substrate (*p* for trend = 0.0121) (Figure 2D) were more frequent in the more favorable response groups. Formal assessment of the proportional-odds assumption indicated some evidence of departure from proportional odds (global *p* = 0.017). In threshold-specific cumulative logistic sensitivity analyses, the direction of the principal baseline associations was generally consistent across the ordered response transitions, although the magnitude of some associations varied between thresholds. Accordingly, the pooled ordinal odds ratios are interpreted as summary associations across the ordered response phenotype rather than as strictly constant effects at each response threshold. Assessment of nonlinear relationships showed evidence of nonlinearity for age (*p* < 0.001), but not for baseline LVEF (*p* = 0.311) or baseline QRS duration (*p* = 0.099). In a sensitivity analysis allowing for a nonlinear age effect, the direction of the principal associations remained consistent, although the association with dilated cardiomyopathy was attenuated to borderline significance (*p* = 0.053). VIFs ranged from 1.08 to 3.90, indicating no evidence of problematic multicollinearity.

Permutation-importance analysis showed that the strongest pre-implant contributors were baseline LBBB, baseline LVEF, and female sex, followed by absence of bundle branch block, absence of atrial fibrillation, and dilated substrate. These findings were directionally consistent with the ordinal regression results. Taken together, these findings suggest that baseline phenotype captures clinically meaningful but only limited variability in subsequent response class, supporting its role as a modest stratification tool rather than a stand-alone predictor of reverse-remodeling outcome. In a sensitivity analysis excluding patients whose LVESV reduction was within ±1 percentage point of the prespecified response thresholds, the analytic sample was reduced from 334 to 259 patients. The direction of the principal baseline associations remained consistent with the primary analysis, although statistical precision was reduced in the smaller sample.

### 3.3. Peri-Implant Analysis: Model 2

For the peri-implant analysis, Model 2 combined the out-of-fold pre-implant probabilities of negative response, nonresponse, response, and super-response generated by Model 1 with prespecified peri-implant variables. Relative to Model 1 in the same analytic subset, Model 2 showed inferior overall performance, with an approximately 4.3% lower weighted one-vs-rest ROC-AUC and an 8.6% lower balanced accuracy. Thus, although peri-implant variables carried clinically relevant information, their addition did not materially enhance cohort-level classification beyond the baseline pre-implant framework. The proportional-odds assumption was not rejected for Model 2 (global *p* = 0.738), and no significant nonlinear relationships were identified for its continuous predictors (all *p* ≥ 0.115). Collinearity diagnostics were also acceptable, with factor-adjusted generalized VIFs ≤1.11. Additional out-of-fold performance assessment showed multiclass Brier scores of 0.741 for Model 1 and 0.749 for Model 2. Class-specific one-vs-rest ROC-AUCs ranged from 0.569 to 0.605 for Model 1 and from 0.516 to 0.667 for Model 2. Full class-specific precision, recall, F1 scores, calibration measures, and confusion matrices are provided in the Supplementary Material.

In the stacked ordinal model used for interpretability, ΔQRS emerged as the strongest peri-implant correlate of higher final response class (OR: 1.41; 95% CI: 1.15–1.73; *p* = 0.0008). By contrast, RV1SI post-implant ECG pattern, LV lead position, and LV lead depth were not significantly associated with response class in the final stacked ordinal model (Table 3). The pre-implant probability of super-response and the pre-implant probability of response also remained independently associated with higher final response class (OR: 1.39; 95% CI: 1.03–1.86; *p* = 0.030; and OR: 1.27; 95% CI: 1.01–1.59; *p* = 0.043, respectively). Permutation-importance analysis similarly ranked ΔQRS as the most influential raw peri-implant feature, followed by the pre-implant probability terms, with more limited contribution from RV1SI post-implant ECG pattern, LV lead position, and LV lead depth. Consistent with the descriptive trend across response classes (*p* for trend = 0.0004) (Figure 2E), Figure 3 showed that increasing ΔQRS was associated with a lower estimated probability of negative response (Figure 3A) and a higher estimated probability of super-response (Figure 3D) across the full cohort, whereas the nonresponder (Figure 3B) and responder (Figure 3C) classes showed more modest intermediate gradients across the ΔQRS spectrum.

Although Model 2 did not improve overall predictive performance, exploratory stratification by baseline predicted response probability showed a numerical improvement in the intermediate-probability tertile. In Figure 4A, Model 2 showed numerically higher discrimination for positive outcome than Model 1 in the intermediate stratum, whereas corresponding improvement was not observed in the low- or high-probability strata. Figure 4B similarly showed numerical increases in the displayed performance metrics within the intermediate group, with no corresponding improvement in the low- or high-probability groups. These findings should be considered exploratory and hypothesis-generating rather than evidence of established subgroup-specific predictive benefit, particularly in the absence of external validation. Independent validation is required to determine whether peri-implant information provides meaningful predictive refinement in patients with intermediate baseline-predicted response probability.

Confidence intervals for between-model performance differences and formal interaction testing across baseline-probability strata were not performed; therefore, these subgroup findings are descriptive and hypothesis-generating and should not be interpreted as evidence of subgroup-specific incremental clinical utility.

### 3.4. Post-Landmark Clinical Outcomes According to Response Phenotype

Table 4 summarizes the post-landmark clinical outcomes across the four reverse-remodeling response groups. A consistent gradient was observed across major endpoints, with super-responders showing the most favorable subsequent clinical course and negative responders the least favorable. This pattern was evident for all-cause mortality, first heart failure hospitalization, and first appropriate ICD shock, whereas advanced heart failure intervention endpoints (LVAD/HTx) were infrequent and were therefore interpreted descriptively. The detailed time-to-event and competing-risk analyses for each endpoint are presented below.

### 3.5. All-Cause Mortality

For all-cause mortality, Kaplan–Meier analysis from the first post-CRT echocardiographic assessment demonstrated significant separation across the four LVESV response groups (log-rank *p* < 0.001), with super-responders showing the most favorable survival and negative responders the poorest (Figure 5). In the adjusted Cox model, negative responder status remained independently associated with higher all-cause mortality compared with super-responders (HR: 4.41; 95% CI: 1.82–10.70; *p* = 0.0010), whereas nonresponders and responders showed directionally higher but not statistically significant risk estimates.

### 3.6. First Heart Failure Hospitalization

For first HF hospitalization, cumulative incidence increased progressively across less favorable response categories, from 2.9% in super-responders to 9.1% in responders, 13.7% in nonresponders, and 23.3% in negative responders at 60 months (Figure 6). In the parsimonious adjusted cause-specific Cox model, negative responders and nonresponders remained at significantly higher risk than super-responders, whereas responders showed a numerically higher but not statistically significant risk estimate. Higher baseline LVEF was independently associated with lower cause-specific risk of first HF hospitalization. However, only 33 post-landmark heart failure hospitalization events were observed, and the corresponding hazard-ratio estimates had wide confidence intervals. These estimates should therefore be interpreted primarily as evidence of a graded risk pattern across remodeling phenotypes rather than as precise measures of effect magnitude.

### 3.7. First Appropriate ICD Shock

For first appropriate ICD shock, cumulative incidence increased progressively across less favorable response categories, reaching 29.3% in negative responders, 24.3% in nonresponders, 16.9% in responders, and 9.0% in super-responders at 60 months (Figure 7). In the parsimonious adjusted cause-specific Cox model, negative responders (HR: 3.88; 95% CI: 1.56–9.66; *p* = 0.0036) and nonresponders (HR: 2.66; 95% CI: 1.08–6.55; *p* = 0.0330) remained at significantly higher risk than super-responders, whereas responders showed only a directional increase that did not reach statistical significance. Higher baseline NYHA class was independently associated with greater cause-specific risk of first appropriate ICD shock.

### 3.8. Low-Count Advanced Heart Failure Intervention Endpoints

For the combined endpoint of first LVAD implantation or heart transplantation after the landmark, the estimated 60-month cumulative incidence was 4.6% in negative responders, 1.2% in nonresponders, 3.2% in responders, and 0.0% in super-responders. Because only nine post-landmark events were observed overall (three LVAD-first and six HTx-first), these advanced heart failure intervention outcomes were summarized descriptively and not modeled further.

## 4. Discussion

A primary challenge in cardiac resynchronization therapy (CRT) remains the lack of consensus regarding response definitions. With more than 17 distinct criteria utilized in the literature, binary responder/nonresponder labels may not fully capture the heart’s biological continuum. The current analysis utilizes a four-class LVESV framework to account for the biological heterogeneity emphasized by Fornwalt et al. [4] and Gold et al. [6]. By distinguishing between nonresponders and a high-risk “negative responder” group, this approach provides a granular risk stratification tool that reflects divergent long-term trajectories.

Our baseline predictor profile largely reproduces the substrate-response paradigm established in MADIT-CRT [8] and refined in subsequent subanalyses [15]. In our cohort, female sex, native LBBB, dilated/nonischemic cardiomyopathy, and higher baseline LVEF were each associated with more favorable reverse remodeling, reinforcing the central role of myocardial substrate and electrical phenotype in shaping CRT response. Importantly, contemporary studies have continued to confirm the predictive relevance of female sex [16,17], LBBB [17], nonischemic substrate [17], and higher baseline LVEF [17] across diverse practice settings, underscoring that these associations are not confined to the original landmark datasets but remain reproducible in modern CRT populations many years later.

Beyond baseline substrate, our data identify ΔQRS narrowing as a peri-implant correlate of more favorable remodeling. This observation is concordant with prior work linking greater post-CRT QRS shortening to reverse remodeling and improved outcomes. Rickard et al. [12] demonstrated that QRS narrowing after CRT was independently associated with enhanced reverse remodeling after adjustment for baseline QRS duration and QRS morphology, whereas more contemporary studies [18] have suggested that acute post-implant QRS narrowing after CRT is independently associated with improved long-term clinical outcome, supporting the concept that early electrical shortening may reflect more effective resynchronization. However, direct numerical comparison should be interpreted cautiously, because prior studies generally used binary response definitions and, in some cases, threshold-based or indexed QRS metrics, whereas our analysis modeled ΔQRS as a continuous predictor across an ordinal four-class remodeling phenotype. Although peri-implant information did not improve overall predictive performance, exploratory stratification suggested a numerical improvement within the intermediate baseline-probability stratum. This observation should be considered hypothesis-generating and does not establish subgroup-specific predictive benefit. ΔQRS remained the strongest peri-implant correlate of favorable remodeling in the full cohort, but independent validation is required to determine whether peri-implant electrical information provides clinically meaningful refinement among patients with intermediate baseline-predicted response probability.

Effective CRT delivery is influenced by several factors that were not systematically captured in the present retrospective dataset, including achieved effective biventricular pacing percentage, device programming, AV/VV optimization, ventricular ectopic burden, myocardial scar extent and location, and the spatial relationship between the LV lead and scar. These factors may influence both acute electrical response and subsequent reverse remodeling and may therefore contribute to residual confounding in the observed association between ΔQRS narrowing and remodeling phenotype. In particular, myocardial scar may limit the mechanical response to pacing despite apparent electrical resynchronization, whereas suboptimal lead–scar relationships, programming, or incomplete effective biventricular capture may attenuate both QRS narrowing and reverse remodeling. These considerations may be particularly relevant in patients with atrial fibrillation. Permanent AF can interfere with effective CRT delivery because irregular intrinsic ventricular activation and competition with paced activation may reduce effective biventricular capture. The clinical impact of AF in CRT therefore depends not only on rhythm classification itself but also on the degree of achieved effective pacing and the accompanying rate- or rhythm-control strategy. Consequently, the observed associations involving AF should be interpreted cautiously and cannot distinguish the effect of AF itself from that of incomplete CRT delivery.

Myocardial deformation imaging may provide complementary phenotypic information beyond conventional LVEF and LVESV. Speckle-tracking-derived LV global longitudinal strain can identify abnormalities in myocardial deformation and may contribute to the characterization of adverse myocardial substrate and tissue alterations associated with fibrosis, although strain abnormalities are not specific for fibrosis and should not be considered a direct surrogate for fibrosis assessed by cardiac magnetic resonance or histology [19]. This may be particularly relevant in CRT candidates, in whom myocardial fibrosis and scar burden can influence electrical propagation, mechanical resynchronization, and subsequent reverse remodeling. Recent evidence in patients undergoing CRT-D further suggests that baseline LV-GLS may provide prognostic information regarding subsequent heart failure outcomes and echocardiographic response. In a cohort of 143 CRT-D recipients, higher absolute baseline GLS values were observed among patients remaining free from HF-related death or hospitalization and also differed between subsequent CRT responders and nonresponders [20]. Because myocardial strain measurements were not systematically available in the present cohort, their potential complementary contribution could not be assessed. Future prospective studies integrating baseline and serial LV-GLS with graded LVESV-based remodeling phenotypes may determine whether myocardial deformation provides additional prognostic information beyond conventional clinical, electrical, and volumetric parameters. Myocardial work may further complement conventional and strain-based assessment by providing additional information on mechanical dyssynchrony, CRT response, and prognosis; however, myocardial work indices were not available in the present cohort and warrant prospective evaluation [21].

The clinical relevance of this graded remodeling taxonomy was further reinforced by the post-landmark hard-outcome analysis. Rather than behaving as a purely echocardiographic construct, response phenotype separated subsequent mortality, first heart failure hospitalization, and first appropriate ICD shock in a coherent gradient, with super-responders showing the most favorable trajectory and negative responders the least favorable. For heart failure hospitalization in particular, the adjusted hazard-ratio estimates were accompanied by wide confidence intervals, reflecting the limited number of events and substantial statistical imprecision. Accordingly, these estimates are more appropriately interpreted as supporting the presence of a graded risk pattern across remodeling phenotypes than as precise estimates of the magnitude of relative risk. This pattern is concordant with prior work indicating that the magnitude of reverse remodeling after CRT carries direct prognostic significance. Ypenburg et al. showed that greater LV reverse remodeling after CRT was associated with less heart failure hospitalization and lower mortality during follow-up, with the most favorable hospitalization-free survival observed among super-responders and the least favorable among negative responders [5], while pooled prospective data later showed that both improved and stabilized patients have lower mortality than worsened patients, supporting graded rather than dichotomous response classification [22]. The arrhythmic component of our findings is likewise aligned with MADIT-CRT. In one analysis, each 10% reduction in LVESV was associated with an approximately 20% lower risk of subsequent ventricular tachyarrhythmia [23]. In a separate LVEF-normalization analysis, patients who achieved post-CRT LVEF >50% had very low arrhythmic risk together with a lower risk of HF/death than patients with persistent severe systolic dysfunction [24]. Importantly, our data also support the clinically relevant concept that favorable remodeling attenuates, but does not abolish, the arrhythmic substrate. Prior series have shown that super-responders experience lower rates of appropriate ICD therapy [25], although arrhythmic risk is not completely abolished even in this subgroup [26]. More broadly, this inverse relationship between echocardiographic response and ventricular arrhythmic burden is supported by meta-analytic data demonstrating lower ventricular arrhythmia risk among CRT responders than among nonresponders [27]. Taken together, these observations reinforce the prognostic importance of early reverse remodeling after CRT-D, particularly for identifying patients who remain at increased risk for subsequent mortality, heart failure events, and ventricular arrhythmic burden, despite device therapy.

At this point, several limitations should be acknowledged. This was a retrospective, single-center study and is therefore subject to selection bias, unmeasured confounding, and limited external generalizability. In addition, patient selection, follow-up intensity, echocardiographic timing, and device management reflected real-world practice at a tertiary referral center rather than a protocolized trial setting, which enhances clinical relevance but may reduce comparability with landmark cohorts. Because inclusion required baseline and follow-up echocardiography and complete device follow-up at our center, patients with very early adverse events, incomplete surveillance, or follow-up partly outside our institution may be underrepresented. The four-class LVESV framework was intentionally chosen to preserve the biologic gradient of reverse remodeling, but it reflects a single echocardiographic domain assessed at the first eligible follow-up within 1 year and does not fully capture other clinically relevant dimensions of CRT response, including symptoms, biomarkers, exercise capacity, quality of life, or longer-term remodeling; variable timing of follow-up echocardiography may also have introduced some misclassification, particularly in patients with delayed remodeling. In addition, echocardiographic measurements were derived from routine clinical reports rather than from a standardized study-specific core-laboratory re-analysis, and formal intraobserver and interobserver reproducibility data were unavailable. The method used for LV volume calculation could not be reliably reconstructed for all examinations across the study period. Measurement variability may therefore have resulted in phenotype misclassification, particularly for patients with LVESV changes close to the prespecified 0%, 15%, and 30% thresholds. Although exclusion of patients immediately adjacent to these thresholds did not reverse the direction of the principal associations, this sensitivity analysis cannot exclude measurement-related misclassification. Myocardial strain measurements, including LV-GLS, were not systematically available across the study period and therefore could not be incorporated into the echocardiographic phenotype or predictive analyses. Their absence limits assessment of whether myocardial deformation provides complementary prognostic information beyond conventional LV volumetric measures. Model discrimination was modest and only internally validated, so the predictive analyses should be viewed as explanatory and hypothesis-generating rather than ready for routine clinical application. Uncertainty from the first-stage Model 1 probability estimates was not formally propagated into the stacked Model 2. As mentioned before, several important determinants of effective CRT delivery were not systematically captured. These unmeasured factors may have influenced both ΔQRS narrowing and reverse remodeling and therefore represent potential sources of residual confounding; accordingly, the association between ΔQRS narrowing and favorable remodeling should be interpreted as correlational rather than causal. The relatively limited number of heart failure hospitalization events also resulted in wide confidence intervals for the adjusted hazard-ratio estimates, limiting precision regarding the magnitude of between-group differences. Finally, some peri-implant variables were represented by relatively sparse categories, the landmark design necessarily excluded pre-landmark events and did not capture total risk from implant, and event counts were limited for some secondary outcomes, especially LVAD implantation and heart transplantation, restricting those analyses to descriptive reporting or parsimonious adjustment.

In conclusion, after CRT-D, early reverse-remodeling phenotype identifies clinically distinct post-landmark risk profiles. Baseline substrate influences the likelihood of favorable remodeling, peri-implant electrical response provides additional mechanistic information but did not improve overall predictive performance in the present cohort, and less favorable remodeling is associated with progressively higher risks of mortality, heart failure hospitalization, and appropriate ICD shock. Together, these findings support early reverse remodeling as a clinically meaningful integrative marker of subsequent outcome after CRT-D.

## Figures and Tables

**Figure 1:**
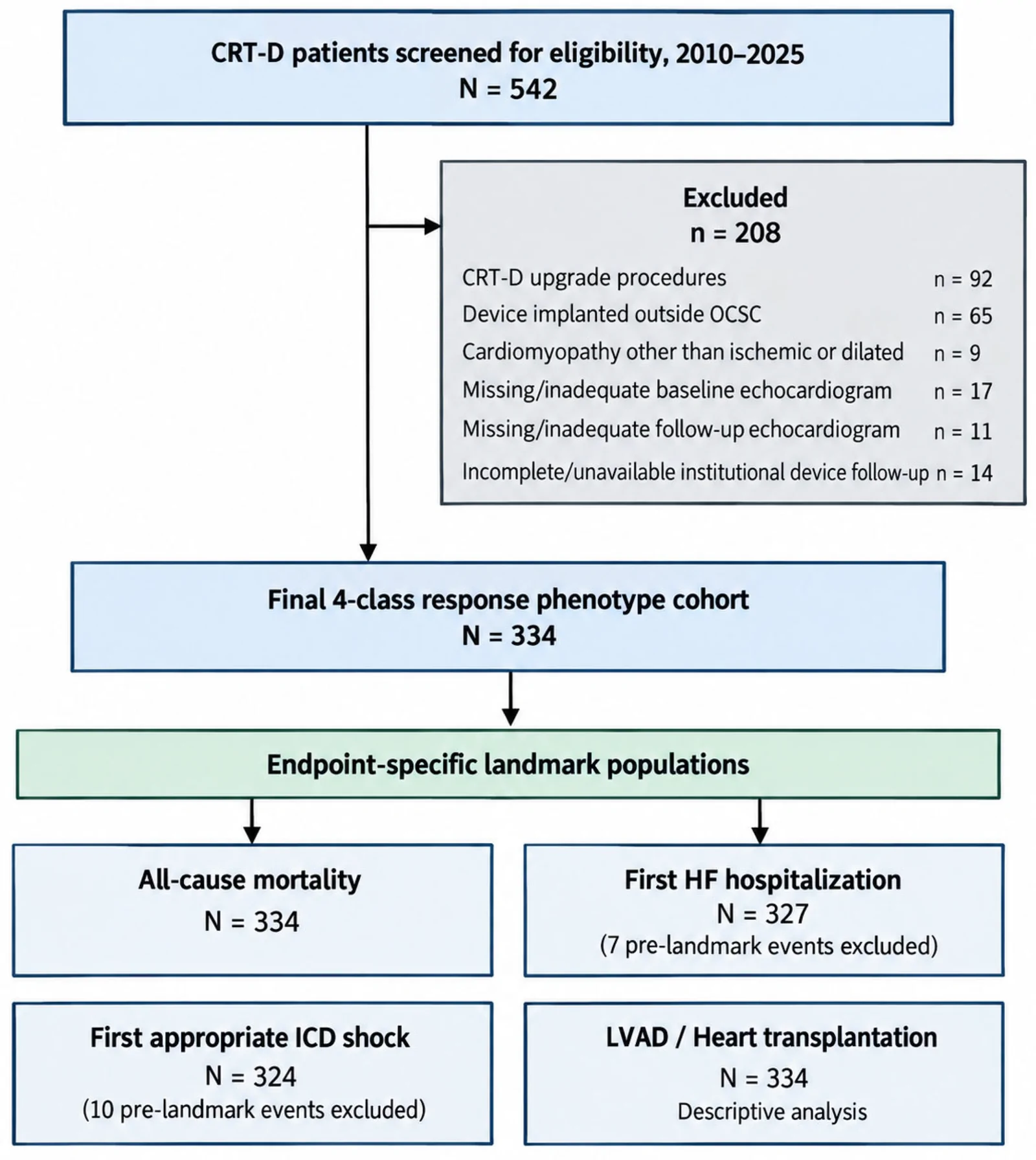
Patient selection and endpoint-specific analytic populations. Flow diagram showing derivation of the final 334-patient CRT-D cohort from 542 screened patients and the endpoint-specific post-landmark populations. **Abbreviations:** CRT-D, cardiac resynchronization therapy with defibrillator backup; HF, heart failure; ICD, implantable cardioverter-defibrillator; LVAD, left ventricular assist device; OCSC, Onassis Cardiac Surgery Center.

**Figure 2:**
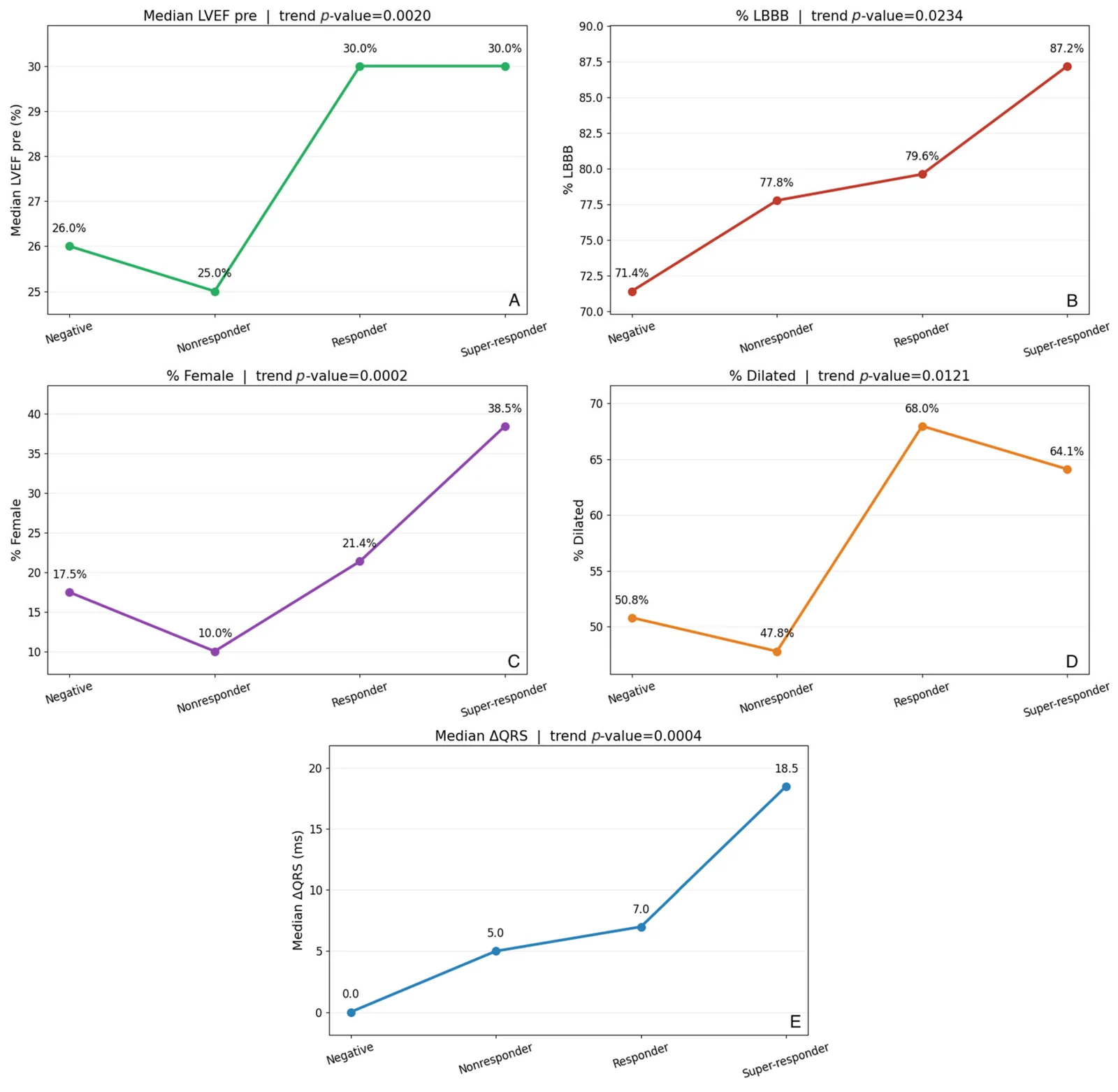
Baseline and peri-implant trends across response phenotypes. Baseline and peri-implant trends across the 4 echocardiographic response phenotypes after CRT-D. More favorable remodeling was associated with higher baseline LVEF (**A**), greater prevalence of LBBB (**B**), greater representation of women (**C**), more frequent dilated cardiomyopathy (**D**), and greater ΔQRS narrowing (**E**), consistent with the ordinal regression findings. **Abbreviations**: CRT-D = cardiac resynchronization therapy with defibrillator backup; LBBB = left bundle branch block; LVEF = left ventricular ejection fraction.

**Figure 3:**
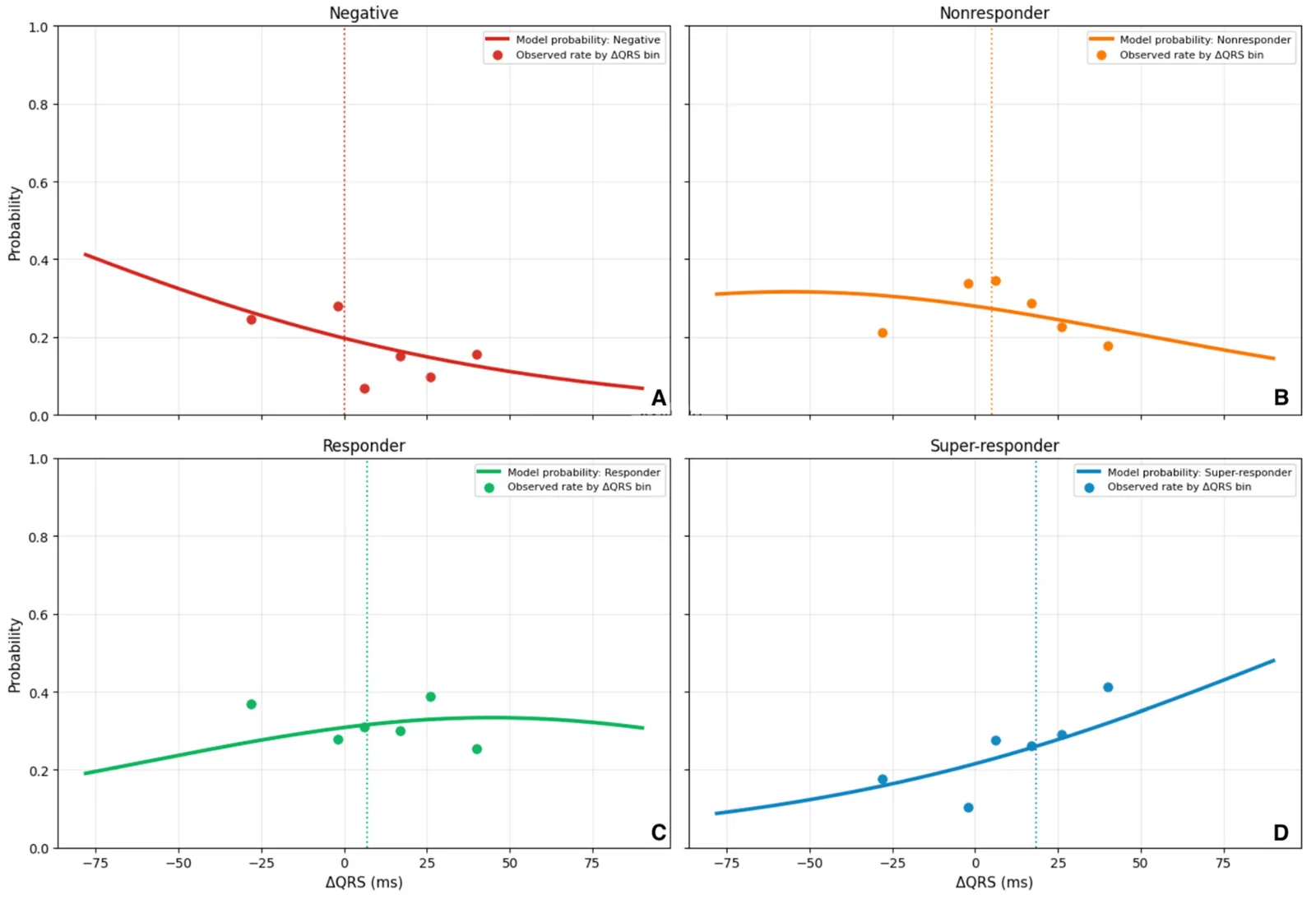
ΔQRS and modeled response probabilities. Association between ΔQRS and modeled response probabilities in the full analytic cohort. Panels show negative response (**A**), nonresponse (**B**), response (**C**), and super-response (**D**). Solid lines represent class-specific modeled probabilities, circles observed frequencies within ΔQRS bins, and vertical dotted lines median ΔQRS by response class. Greater QRS narrowing was associated with higher probability of favorable remodeling. **Abbreviations**: CRT-D = cardiac resynchronization therapy with defibrillator backup.

**Figure 4:**
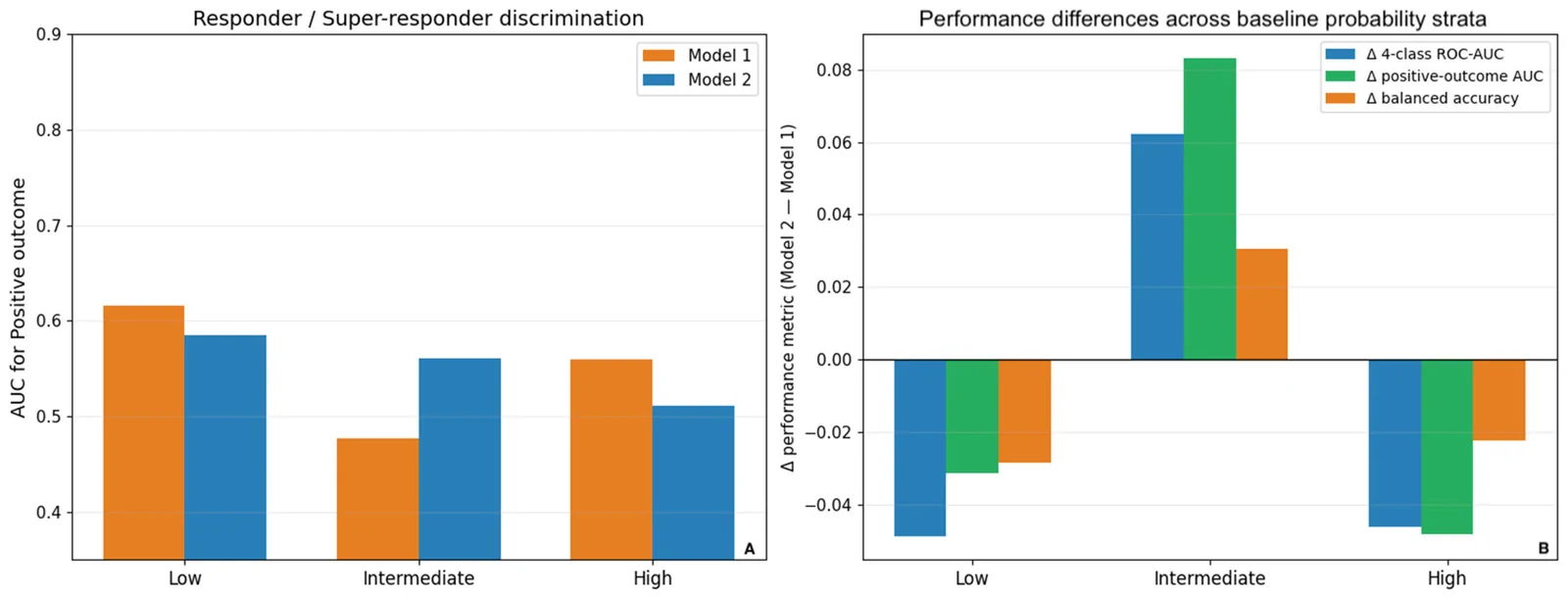
Exploratory model performance across baseline probability strata. This figure presents an exploratory comparison of Model 1 and Model 2 performance across tertiles of baseline predicted positive-response probability. Panel (**A**) compares discrimination for responder or super-responder status between Model 1 and Model 2. Panel (**B**) shows the corresponding differences in 4-class ROC-AUC, positive-outcome AUC, and balanced accuracy. Numerical improvement with Model 2 was observed in the intermediate-probability stratum, whereas corresponding improvement was not observed in the low- or high-probability strata. These subgroup findings should be considered exploratory and hypothesis-generating and require external validation. **Abbreviations**: AUC = area under the receiver operating characteristic curve; ROC = receiver-operating characteristic.

**Figure 5:**
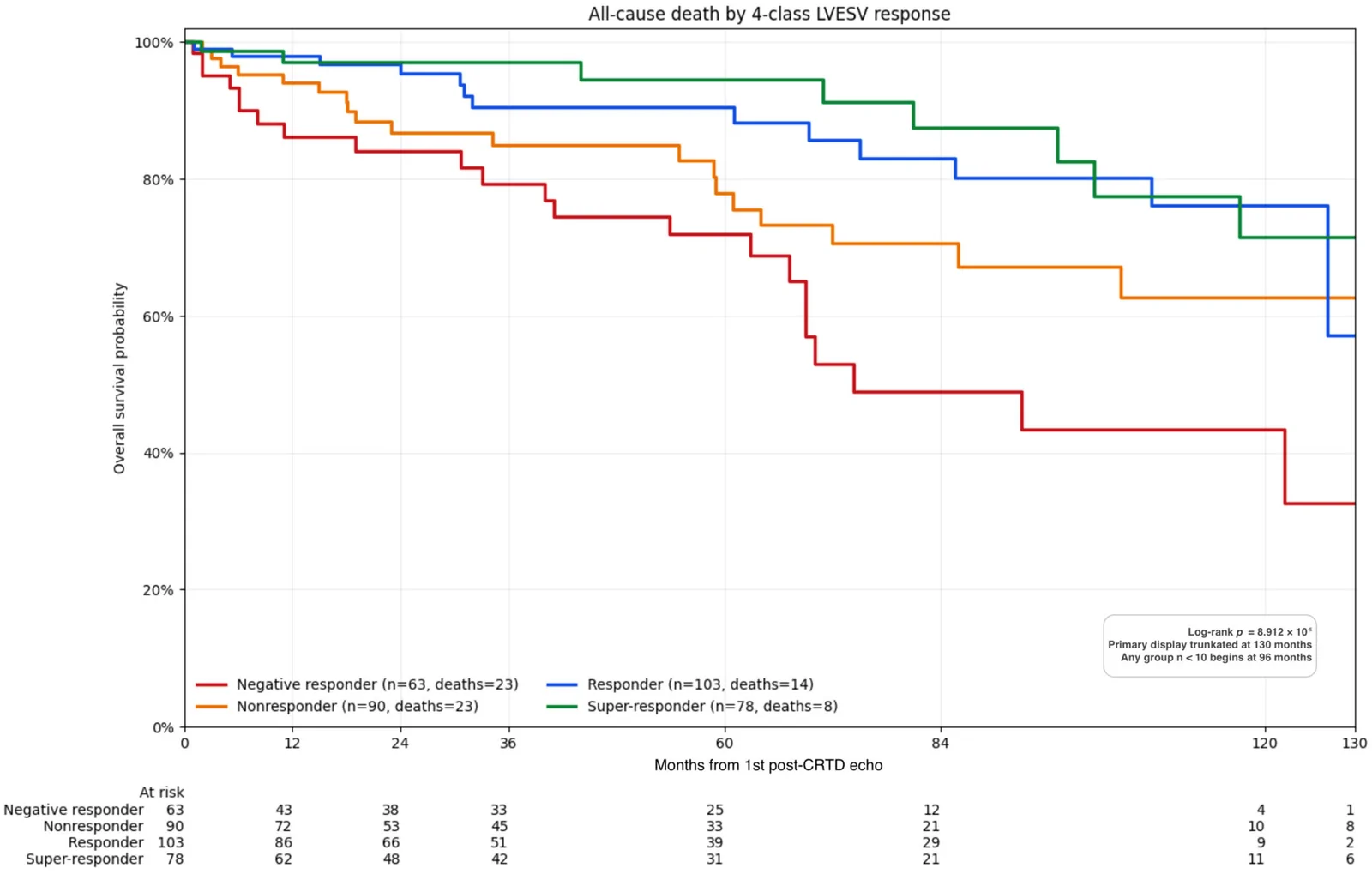
All-cause mortality by response phenotype. This Kaplan–Meier figure shows all-cause survival according to the 4-class LVESV response phenotype, with time zero defined at the first post-CRT echocardiographic assessment. Survival separated progressively across groups, with super-responders showing the most favorable survival and negative responders the poorest outcome, supporting early reverse remodeling as a marker of subsequent mortality risk after CRT-D. **Abbreviations**: CRT-D = cardiac resynchronization therapy with defibrillator backup; LVESV = left ventricular end-systolic volume.

**Figure 6:**
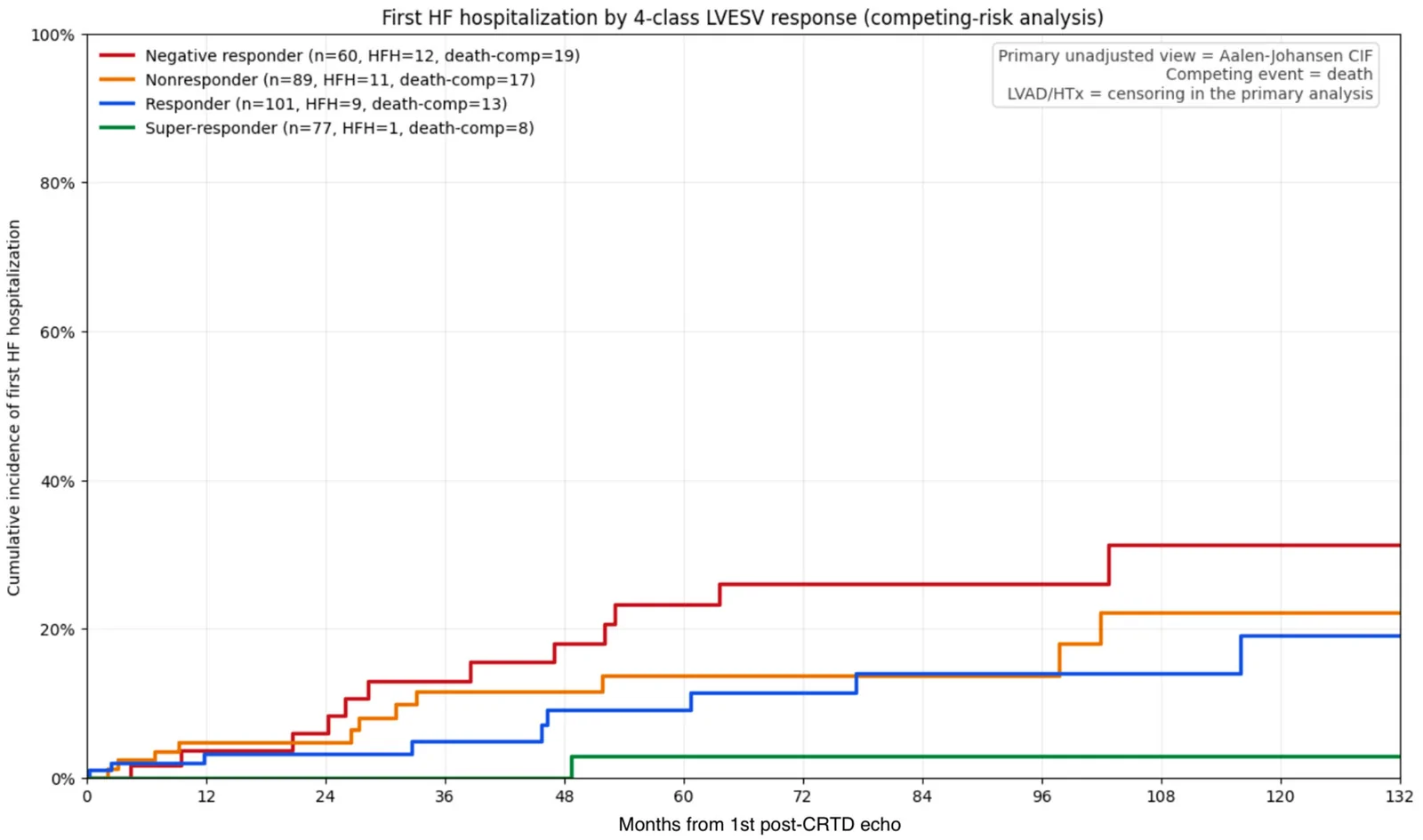
First heart failure hospitalization by response phenotype. This figure shows the cumulative incidence of first heart failure hospitalization after landmark response classification according to the 4-class LVESV phenotype. Estimates used competing-risk methods, with death treated as the competing event and LVAD implantation or heart transplantation as censoring events. Less favorable remodeling was associated with progressively higher post-landmark heart failure hospitalization burden. **Abbreviations**: HF = heart failure; HTx = heart transplantation; LVAD = left ventricular assist device; LVESV = left ventricular end-systolic volume.

**Figure 7:**
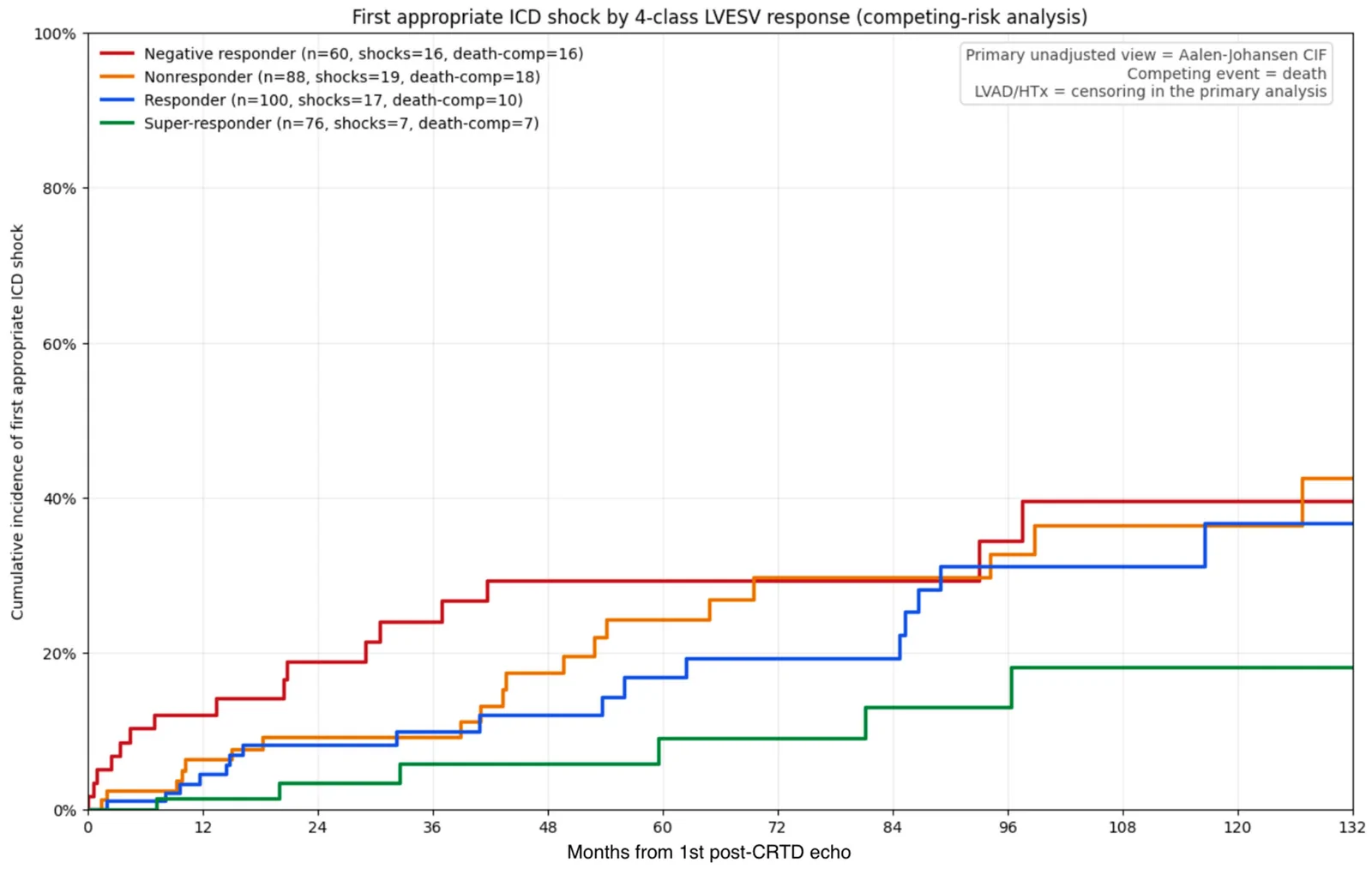
First appropriate ICD shock by response phenotype. This figure shows the cumulative incidence of first appropriate ICD shock after landmark response classification according to the 4-class LVESV phenotype. Estimates used competing-risk methods, with death treated as the competing event and LVAD implantation or heart transplantation as censoring events. Less favorable remodeling was associated with progressively higher arrhythmic event burden after CRT-D. **Abbreviations**: HTx = heart transplantation; ICD = implantable cardioverter-defibrillator; LVAD = left ventricular assist device; LVESV = left ventricular end-systolic volume.

**Table 1:** Baseline characteristics of the overall CRT-D cohort. Baseline demographic, clinical, electrocardiographic, and echocardiographic characteristics of the study population are shown for the overall analytic cohort. Continuous variables are presented as mean ± SD and categorical variables as *n* (%). Heart failure substrate, comorbidities, atrial fibrillation status, baseline conduction pattern, and New York Heart Association functional class are summarized at the time of CRT-D evaluation.

Characteristic	Overall Cohort (*N* = 334)
Age, y	65.3 ± 11.1
Female sex	72 (21.6)
***Heart failure substrate***	
Ischemic cardiomyopathy	139 (41.6)
Dilated cardiomyopathy	195 (58.4)
***Comorbities***	
Hypertension	281 (84.1)
Diabetes mellitus	118 (35.3)
Hyperlipidemia	231 (69.2)
Prior PCI	75 (22.5)
Prior CABG	58 (17.4)
***Atrial fibrillation status***	
None	160 (47.9)
Permanent	79 (23.7)
Paroxysmal	95 (28.4)
***Baseline conduction pattern***	
LBBB	265 (79.3)
RBBB	17 (5.1)
No bundle branch block	52 (15.6)
QRS duration before implantation, ms	157.6 ± 25.3
***LVEF before CRT-D, %***	28.8 ± 5.4
***NYHA functional class***	
II	34 (10.2)
III	282 (84.4)
IV	18 (5.4)

**Abbreviations**: CABG = coronary artery bypass grafting; CRT-D = cardiac resynchronization therapy with defibrillator backup; LBBB = left bundle branch block; LVEF = left ventricular ejection fraction; NYHA = New York Heart Association; PCI = percutaneous coronary intervention; RBBB = right bundle branch block; SD = standard deviation.

**Table 2:** Model 1: pre-implantation multivariable ordinal model for higher 4-class echocardiographic response phenotype after CRT-D. Ordinal logistic regression of pre-implant clinical and electrocardiographic variables associated with the 4-class echocardiographic response phenotype after cardiac resynchronization therapy with defibrillator backup.

Variable	β Coefficient	SE	z Value	Adjusted OR	95% CI	*p* Value
Female sex	0.302	0.108	2.783	1.35	1.09–1.67	***0.005***
Baseline LVEF	0.232	0.108	2.139	1.26	1.02–1.56	***0.032***
LBBB	0.362	0.175	2.077	1.44	1.02–2.02	***0.038***
Dilated cardiomyopathy substrate	0.216	0.109	1.978	1.24	1.00–1.54	***0.048***
No atrial fibrillation	0.226	0.133	1.700	1.25	0.97–1.63	0.089
Paroxysmal atrial fibrillation	0.174	0.129	1.349	1.19	0.92–1.53	0.177
No bundle branch block	0.219	0.189	1.159	1.24	0.86–1.80	0.246
Age	0.125	0.112	1.111	1.13	0.91–1.41	0.267
QRS duration	−0.057	0.115	−0.495	0.94	0.75–1.18	0.620

**Footnotes:** Odds ratios represent the odds of belonging to a higher echocardiographic response class. Positive β coefficients and adjusted odds ratios >1 indicate greater odds of a more favorable response phenotype. **Abbreviations:** CRT-D = cardiac resynchronization therapy with defibrillator backup; LBBB = left bundle branch block; LVEF = left ventricular ejection fraction; OR = odds ratio; SE = standard error.

**Table 3:** Model 2: peri-implantation correlates of higher 4-class echocardiographic response phenotype after CRT-D. Ordinal logistic regression of peri-implantation electrical and lead-related variables associated with a higher echocardiographic response class after cardiac resynchronization therapy with defibrillator backup.

Variable	β Coefficient	SE	z Value	Adjusted OR	95% CI	*p* Value
ΔQRS	0.347	0.104	3.344	1.41	1.15–1.73	***<0.001***
Positive V1/negative I pattern	0.123	0.100	1.234	1.13	0.93–1.38	0.217
Midlateral lead position	−0.155	0.106	−1.458	0.86	0.70–1.05	0.145
Posterolateral lead position	−0.090	0.116	−0.779	0.91	0.73–1.15	0.436
Epicardial lead position	−0.022	0.100	−0.224	0.98	0.80–1.19	0.823
Basal LV lead depth	−0.215	0.226	−0.949	0.81	0.52–1.26	0.343
Mid LV lead depth	−0.107	0.210	−0.508	0.90	0.59–1.36	0.611

**Footnotes:** Odds ratios represent the odds of belonging to a higher echocardiographic response class. ΔQRS is expressed per 10 ms greater QRS narrowing and was calculated as pre-implant QRS duration minus post-implant QRS duration. Reference categories were anterolateral LV lead position for lead position and apical LV lead depth for lead depth. Model 2 additionally included out-of-fold pre-implant predicted probabilities from Model 1 as stacked model-derived terms. This table displays the raw peri-implant electrical and lead-related variables for clinical interpretability. Positive β coefficients and adjusted odds ratios >1 indicate greater odds of a more favorable response phenotype. **Abbreviations:** CRT-D = cardiac resynchronization therapy with defibrillator backup; LV = left ventricular; OR = odds ratio; SE = standard error.

**Table 4:** Post-landmark clinical outcomes according to 4-class echocardiographic response phenotype after CRT-D. Post-landmark outcome analysis across the 4 echocardiographic response groups after cardiac resynchronization therapy with defibrillator backup. The table summarizes event burden, 60-month cumulative incidence for selected nonfatal outcomes, and adjusted hazard ratios using the super-responder group as the reference category.

Endpoint	Total Events/ Risk Set	Negative Responder	Nonresponder	Responder	Super- Responder	Adjusted HR (95% CI), Reference = Super-Responder
All-cause mortality	68/334	23/63 deaths	23/90 deaths	14/103 deaths	8/78 deaths	4.41 (1.82–10.70), *p* = 0.001; 2.40 (0.95–6.05), *p* = 0.064; 2.16 (0.82–5.65), *p* = 0.118
First appropriate ICD shock	59/324	29.3% at 60 months	24.3% at 60 months	16.9% at 60 months	9.0% at 60 months	3.88 (1.56–9.66), *p* = 0.0036; 2.66 (1.08–6.55), *p* = 0.033; 2.19 (0.89–5.37), *p* = 0.087
First heart failure hospitalization	33/327	23.3% at 60 months	13.7% at 60 months	9.1% at 60 months	2.9% at 60 months	17.79 (2.29–138.19), *p* = 0.0059; 10.62 (1.34–83.91), *p* = 0.025; 7.58 (0.95–60.30), *p* = 0.056
First LVAD implantation or heart transplantation	9/334	5 events	1 event	3 events	0 events	Descriptive analysis only

**Footnotes:** Hazard ratios are presented in the following order: negative responder versus super-responder, nonresponder versus super-responder, and responder versus super-responder. Percentages for first appropriate ICD shock and first heart failure hospitalization represent estimated 60-month cumulative incidence. First LVAD implantation or heart transplantation was reported descriptively because of the low number of events. **Abbreviations:** CI = confidence interval; CRT-D = cardiac resynchronization therapy with defibrillator backup; HR = hazard ratio; ICD = implantable cardioverter-defibrillator; LVAD = left ventricular assist device.

## Data Availability

The data presented in this study are available from the corresponding author upon reasonable request.

## Supplementary Materials

The following supporting information can be downloaded at: https://www.mdpi.com/article/10.3390/jcm15197443/s1. Table S1: Class-specific predictive performance and calibration; Table S2: Out-of-fold confusion matrices.

## Abbreviations

The following abbreviations are used in this manuscript:

CABG	coronary artery bypass grafting
CRT-D	cardiac resynchronization therapy with defibrillator backup
HF	heart failure
HTx	heart transplantation
ICD	implantable cardioverter-defibrillator
LBBB	left bundle branch block
LV	left ventricular
LVAD	left ventricular assist device
LVEF	left ventricular ejection fraction
LVESV	left ventricular end-systolic volume
NYHA	New York Heart Association
PCI	percutaneous coronary intervention
RV1SI	V1-positive/I-negative post-implant electrocardiographic pattern
CI	confidence interval
HR	hazard ratio
OR	odds ratio
ROC-AUC	area under the receiver operating characteristic curve
SE	standard error
