# Supplementary material for: Pre-Implant Substrate, Peri-Implant Electrical Features, and Outcomes Across Four Echocardiographic Phenotypes After CRT-D
Source: J Clin Med. 2026 Sep 24;15(19):7443. doi: 10.3390/jcm15197443 (PMC13665815; doi:10.3390/jcm15197443)
Supplement: Supplementary file 1 [file jcm-15-07443-s001.zip › jcm-4560939-supplementary.pdf]

Supplementary Table S1. Class-specific predictive performance and calibration.

Model	Response class	OvR ROC-AUC	Precision	Recall	F1 score	Brier score	Calibration-in-the-large	Calibration slope
Model 1	Negative responder	0,574	0,273	0,333	0,300	0,156	-0,353	0,648
Model 1	Nonresponder	0,577	0,314	0,356	0,333	0,196	0,116	0,563
Model 1	Responder	0,569	0,385	0,291	0,331	0,215	0,297	0,576
Model 1	Super-responder	0,605	0,390	0,385	0,387	0,174	-0,122	0,727
Model 2	Negative responder	0,555	0,225	0,317	0,263	0,160	-0,368	0,162
Model 2	Nonresponder	0,516	0,284	0,211	0,242	0,203	0,121	0,105
Model 2	Responder	0,517	0,319	0,214	0,256	0,219	0,282	0,181
Model 2	Super-responder	0,667	0,358	0,500	0,417	0,167	-0,098	1,247

Supplementary Table S2. Out-of-fold confusion matrices.

Panel A. Model 1

True class \ Predicted class	Negative	Nonresponder	Responder	Super-responder
Negative responder	**21**	18	14	10
Nonresponder	23	**32**	20	15
Responder	22	29	**30**	22
Super-responder	11	23	14	**30**

Panel B. Model 2

True class \ Predicted class	Negative	Nonresponder	Responder	Super-responder
Negative responder	**20**	18	11	14
Nonresponder	27	**19**	21	23
Responder	28	20	**22**	33
Super-responder	14	10	15	**39**
